# Exosomal circular RNA circ_0074673 regulates the proliferation, migration, and angiogenesis of human umbilical vein endothelial cells via the microRNA-1200/MEOX2 axis

**DOI:** 10.1080/21655979.2021.1967077

**Published:** 2021-09-13

**Authors:** Yan Huang, Bo Liang, Xiangjuan Chen

**Affiliations:** a Obstetrics and Gynecology Department, Shenzhen University General Hospital, Shenzhen, Guangdong, China; b General Surgery Department, Shenzhen University General Hospital, Shenzhen, Guangdong, China

**Keywords:** Gestational diabetes mellitus, human umbilical vein endothelial cells, exosome, circ_0074673, angiogenesis

## Abstract

Circular RNAs (circRNAs) are implicated in the pathogenesis of gestational diabetes mellitus (GDM). The aim of this study was to investigate the roles and molecular mechanism underlying the effects of circ_0074673 in GDM. Exosomal morphology was visualized by transmission electron microscopy (TEM), while exosomal size and concentration were determined by nanoparticle tracking analysis (NTA). The expression of CD9 and CD63 was measured by western blotting. The levels of circ_0074673, miR-1200 and mesenchyme homeobox 2 (MEOX2) were determined by quantitative real-time polymerase chain reaction (qPCR). Cellular proliferation, migration, and angiogenesis were measured by Cell Counting Kit-8 (CCK-8), transwell, and tube formation assays, respectively. The binding relationship between circ_0074673 or MEOX2 and miR-1200 was evaluated by luciferase reporter assay, RNA-binding protein immunoprecipitation (RIP) assay and RNA-pull-down assay. The results showed that exosomal size and concentration were greater in the umbilical cord blood of patients with GDM than in that of the healthy controls. The expression of circ_0074673 was upregulated in exosomes from GDM and in human umbilical vein endothelial cells (HUVECs) co-cultured with exosomes. High glucose (HG) treatment suppressed cellular proliferation, migration, and angiogenesis. Circ_0074673 knockdown enhanced the proliferation, migration, and angiogenesis of HG treated HUVECs (HG-HUVECs). As circ_0074673 and MEOX2 directly bind to miR-1200, circ_0074673 silencing promoted the biological functions of HG-HUVECs by sponging miR-1200 and further targeting MEOX2. Altogether, the loss of exosomal circ_0074673 facilitated the proliferation, migration, and angiogenesis of HG-HUVECs via the miR-1200/MEOX2 axis, suggesting that circ_0074673 is a potential therapeutic target for GDM.

## Introduction

Gestational diabetes mellitus (GDM) is first observed during pregnancy [1], and is related to poor perinatal outcomes, including macrosomia, premature birth, stillbirth, teratogenicity, and neonatal metabolic disorders [2]. On the other hand, pregnant women with GDM have an increased risk of type 2 diabetes mellitus and cardiovascular disease [3]. The incidence of GMD has increased in the recent years. The average morbidity of GDM is approximately 6% [4], and the recurrence rate of GDM ranges from 30% to 84% [5]. The differences in the diagnostic criteria and recommendations for the prevention of GDM employed worldwide make the management of GDM difficult [6]. Furthermore, there is a lack of effective therapeutic strategies against GDM [7]. GDM is closely associated with long-term maternal endothelial dysfunction [8,9]. Persistent endothelial damage increases the risk of developing postpartum cardiovascular diseases. Therefore, the prevention of endothelial dysfunction during pregnancy may provide safe prophylaxis for pregnant women with GDM.

Circular RNAs (circRNAs) are newly discovered non-coding RNAs with a completely stable circular structure [10]. Numerous circRNAs have been implicated in human diseases, including diabetes, neurological diseases, cardiovascular diseases, and malignancies [11]. Accumulating evidence demonstrates that circRNAs play essential roles in the pathogenesis of GDM. The abnormal expression of circRNAs is involved in insulin resistance and β-cell dysfunction, which are the two main features of GDM [12]. The hsa_circ_0074673 is spliced from G3BP1 mRNA, which is located on chromosome 5. However, the role of circ_0074673 in GDM remains to be elucidated.

Exosomes are membrane-bound nano-sized vesicles that transmit biological signals between cells [13], and carry diverse biomolecules, including glycans, lipids, proteins, metabolites, and nucleic acids [14]. Exosomes are emerging as useful biomarkers for the diagnosis, treatment, and prognosis of diseases [15]. A previous study reported an abundance of intact and stable circRNAs in the exosomes in human serum [16]. It has been additionally reported that the expression of circ_0074673 isolated from the exosomes in umbilical cord blood increases in patients with GDM [17]. However, the biological function of exosomal circ_0074673 requires further investigation.

The present study aimed to investigate the effects of exosomal circ_0074673 on the proliferation, migration, and angiogenesis of human umbilical vein endothelial cells (HUVECs) and explore of the underlying molecular mechanism. We found that exosomal circ_0074673 affects the biological behavior of HG-HUVECs by regulating the miR-1200/MEOX2 axis. Thus, the study suggests that circ_0074673 might be a novel therapeutic target for GDM.

## Materials and methods

### Ethics statement

The study protocol was approved by the Ethics Committee of Shenzhen University General Hospital (No. SUGH201806020). Written informed consent was obtained from each participant prior to the study.

### Participants

All the participants were tested for GDM at Shenzhen University General Hospital. A 75-g oral glucose tolerance test (OGTT) was performed at 24–28 weeks of gestation. GDM was defined by fasting plasma glucose levels of 5.1 mmol/l, and/or 1-h plasma glucose levels of ≥10.0 mmol/l, and/or 2-h plasma glucose levels of ≥8.5 mmol/l [18]. The plasma glucose levels of the other participants were normal during the 75-g OGTT. Patients with pre-pregnancy diabetes mellitus, metabolic syndrome, other complications of pregnancy, and infections were excluded from the present study. Samples of umbilical cord blood were collected from the umbilical cord of participants after cesarean delivery (30 samples from patients with GDM, and 30 samples from healthy controls). All the blood samples were stored at −80°C until further analyses. The clinical characteristics of all subjects are displayed in Table 1.

**Table 1.** Clinical characteristics of patients with GDM and healthy controls in this study

Characteristic	GDM (n = 30)	Healthy controls (n = 30)	P value
Age (years)	30.4 ± 3.1	29.6 ± 2.1	0.2467
Pre-gestational BMI (kg/m2)	25.4 ± 3.7	21.9 ± 3.4	0.0003
Hemoglobin (%)	5.5 ± 0.6	4.8 ± 0.4	<0.0001
Fasting plasma glucose (mM)	5.4 ± 0.2	4.3 ± 0.4	<0.0001
One‐hour plasma glucose (mM)	10.8 ± 0.5	6.8 ± 0.3	<0.0001
Two‐hour plasma glucose (mM)	8.6 ± 0.4	5.7 ± 0.4	<0.0001
Gestational age at delivery (wk)	39.0 ± 1.2	39.3 ± 1.2	0.3369
Fetal birth weight (g)	3326 ± 413	3410 ± 382	0.4168

### Isolation and identification of exosomes

Exosomes were isolated from the umbilical cord blood by ultracentrifugation at 4°C. The samples were centrifuged at 3000 × g for 15 min, and the supernatants were centrifuged at 10000 × g for 30 min. After filtering, the supernatants were centrifuged at 100000 × g for 3 h. The pelleted exosomes were resuspended in phosphate-buffered saline (PBS). The exosomes were stored at −80°C until further experimentation.

The morphology of the exosomes was visualized by transmission electron microscopy (TEM). The size and concentration of the exosomes were determined by nanoparticle tracking analysis (NTA).

### Cell culture

HUVECs were purchased from Procell (Wuhan, China). The cells were maintained in endothelial cell growth medium (PromoCell, Germany) in an incubator at 37°C with 5% CO_2_.

The HUVECs in the logarithmic phase were seeded in 24-well plates. When the confluence reached 60%, the cells were co-incubated with the exosomes. The cells were therefore divided into HUVEC and exo-HUVEC groups.

### PKH67 staining

The exosomes were labeled using a PKH67 Green Fluorescent Cell Linker Mini Kit (Sigma-Aldrich, USA), following the manufacturer’s protocol. Briefly, the cells were incubated with 5 μl of PKH67 dye for 4 h. After washing with PBS, the cells were fixed with 4% paraformaldehyde for 30 min and stained with 4ʹ,6-diamidino-2-phenylindole (DAPI) for 5 min. The images were visualized using a fluorescence confocal microscope (Olympus, Japan).

### Cellular treatment

The cells treated with high glucose (HG) were used as an *in vitro* model of GDM. Additional D-glucose (Sigma-Aldrich) was dissolved in the medium until the final concentration of glucose reached 25 mM.

### Cellular transfection

A miR-1200 inhibitor, negative control (NC) inhibitor, miR-1200 mimics, mimics NC, small interfering RNA (si)-NC, and si-circ_0074673 were synthesized by Ribobio (Guangzhou, China). For the study, pcDNA3.1 was purchased from YouBio (Changsha, China), and pcDNA3.1-MEOX2 was structured. HG-HUVACs were seeded into 6-well plates before 24 h of transfection. Lipofectamine 3000 (Invitrogen) was used for transient transfection, according to the supplier’s instructions. The cells were collected after 48 h of transfection for further analysis.

### Cell proliferation analysis

The Cell Counting Kit-8 (CCK-8; Dojindo, Kumamoto, Japan) assay was performed for analyzing cellular proliferation [19]. Briefly, the cells were seeded into 96-well plates and incubated for 0, 12, 24, or 48 h. Next, 10 μl CCK-8 was added to each well and the cells were incubated for 2 h. Finally, the absorbance was measured at 450 nm with a microplate reader (BD Biosciences, USA).

### Cell migration analysis

For the migration assay, 24-well Transwell chambers (8-μm pore size) were used. The cells were placed in the serum-free medium in the upper chambers, and complete medium was added to the lower chambers. After 24 h of incubation, the cells on the upper side of the filters in the upper chambers were removed using cotton swabs. The cells that had invaded and migrated to the other side of the filters were fixed with methanol and stained with 0.1% crystal violet. The number of cells were counted from five randomly selected fields under a microscope (Olympus).

### Tube formation assay

Matrigel (Corning, USA) was diluted with endothelial cell growth medium at a 1:1 ratio. The mixture was then spread on 24-well plates on ice. The transfected cells were seeded into 24-well plates at a density of 5 × 10^4^ cells/well. After 12 h of incubation at 37°C with 5% CO_2_, the images were captured under a bright-field microscope. At least three random fields were selected for analysis [19].

#### Bioinformatic analysis

A target of circ_0074673 was predicted using Circular RNA Interactome online tool (https://circinteractome.irp.nia.nih.gov/), and a target of miR-1200 was predicted using TargetScanHuman 7.2 database (http://www.targetscan.org/vert_72/).

### Luciferase reporter assay

The amplified wild-type (WT) and mutant (MUT) fragments of the 3′-UTR of circ_0074673 and MEOX2 were inserted into pmir-GLO vectors, namely, circ_0074673-WT, circ_0074673-MUT, MEOX2-WT, and MEOX2-MUT. HEK293T cells were co-transfected with WT or MUT plasmids along with miR-1200 mimics or mimics NC in 24-well plates using Lipofectamine 3000. After 48 h of transfection, the luciferase activity was detected using a Dual-Glo Luciferase Assay System (Promega, USA), and calculated by normalizing the activity of firefly luciferase to that of *Renilla* luciferase.

#### RNA-binding protein immunoprecipitation (RIP) assay

Magna RIP™ RNA-Binding Protein Immunoprecipitation Kit (Millipore, Germany) was used to perform RIP assay as previous describe [20]. Briefly, the cells were lysed using RIP lysis buffer with proteinase and RNase inhibitors. The lysates were incubated with anti-AGO2 or anti-IgG conjugated magnetic beads (Millipore) at 4 °C overnight. After washing, the total RNA was extracted and the enrichment was measured using quantitative real-time polymerase chain reaction (qPCR) analysis.

#### RNA pull-down assay

The cells were transfected with biotin labeled miR-1200 or NC for 48 h. Then the cells were lysed using lysis buffer (Ambion, USA). The lysate was incubated with streptavidin magnetic beads (Invitrogen) at 4 °C overnight. After washing, the total RNA was extracted and the enrichment was measured using qPCR analysis [21].

### qPCR analysis

The total RNA was isolated using TRIzol reagent. Reverse transcription (RT) and qPCR were performed using a GoTaq 2-step RT-qPCR system. A Bio-Rad real-time PCR system (Hercules, CA, USA) was used for qPCR, under the following conditions: pre-denaturation at 95°C for 2 min, followed by 40 cycles of denaturation at 95°C for 15 s, and annealing and extension at 60°C for 1 min. Glyceraldehyde-3-phosphate dehydrogenase (GAPDH) and U6 were used for normalization. The expression (fold change) was analyzed using the 2^−ΔΔCt^ method. The specific primers were obtained from Invitrogen.

### Western blotting

The total proteins were extracted from the exosomes and cells using radio immunoprecipitation assay (RIPA) lysis buffer (Sigma-Aldrich). The protein concentration was determined using a bicinchoninic acid (BCA) Protein Assay Kit (Beyotime). Equal amounts of protein were separated using sodium dodecyl sulfate polyacrylamide gel electrophoresis (SDS-PAGE), and subsequently transferred onto polyvinylidene fluoride (PVDF) membranes. The membranes were blocked using 5% nonfat milk and incubated overnight at 4°C with the anti-CD9 and anti-CD63 primary antibodies. The following day, the membranes were incubated for 2 h with horse radish peroxidase (HRP)-conjugated goat anti-rabbit IgG at 25°C room temperature. BeyoECL Plus solution (Beyotime) was used for visualizing the protein bands.

### Statistical analyses

Data from at least three independent experiments were analyzed using GraphPad Prism software, version 6.0 (San Diego, CA, USA), and represented as the mean ± standard deviation (SD). Student’s t-tests were used for comparisons between groups, while comparisons among multiple groups were performed using one-way analysis of variance (ANOVA). Statistical significance was considered at P < 0.05.

## Results

Herein, we aimed to investigate the role of exosomal circ_0074673 in HUVECs. We found that a loss of exosomal circ_0074673 promoted the proliferation, migration and angiogenesis of HG-HUVECs by regulating the miR-1200/MEOX2 axis. Thus, this study may provide novel evidence for circ_0074673 as a target in the treatment for GDM.

### Circ_0074673 levels were upregulated in umbilical cord blood exosomes of patients with GDM

**Figure 1.**
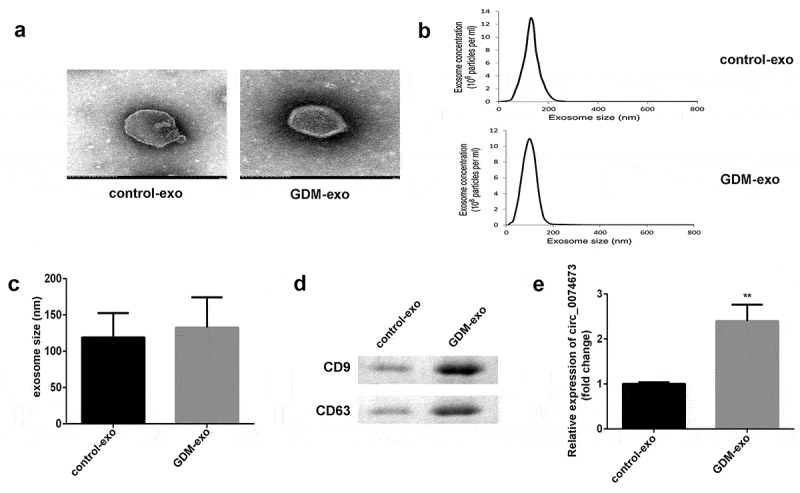
*The expression of circ_0074673 is increased in the exosomes isolated from the umbilical cord blood of patients with GDM*. (a) The morphology of the exosomes was visualized by TEM. (b) The size of the exosomes was detected by NTA. (c) The size of the exosomes was quantified. (d) The levels of CD9 and CD63 were determined by western blotting. (e) The expression of circ_0074673 was detected by qPCR. Each experiment was repeated in three times. **P < 0.01

The exosomes in the umbilical cord blood of patients with GDM and healthy controls were first isolated. The images obtained by TEM revealed the presence of vesicles in the umbilical cord blood of patients with GDM and control subjects (Figure 1(a)). The size and concentration of the exosomes were obtained by NTA (Figure 1(b)). The average size of the exosomes isolated from the patients with GDM (GDM-exo) was larger than that of the healthy controls (control-exo) (Figure 1(c)). The expression of exosomal marker proteins was determined by western blotting. The results demonstrated that the expression of CD9 and CD63 was higher in the exosomes of patients with GDM (GDM-exo) than in those of the healthy controls (control-exo) (Figure 1(d)). The results indicated that the levels of circ_0074673 were significantly higher in the GDM-exo group than in the control-exo group (Figure 1(e)). Moreover, as illustrated in Table 1, there are significant differences in pre-gestational BMI, hemoglobin, fasting plasma glucose, one‐hour plasma glucose and two‐hour plasma glucose. However, there are no significant differences in age, gestational age at delivery and fetal birth weight.

### Circ_0074673 knockdown promoted HG-HUVEC proliferation, migration, and angiogenesis

**Figure 2.**
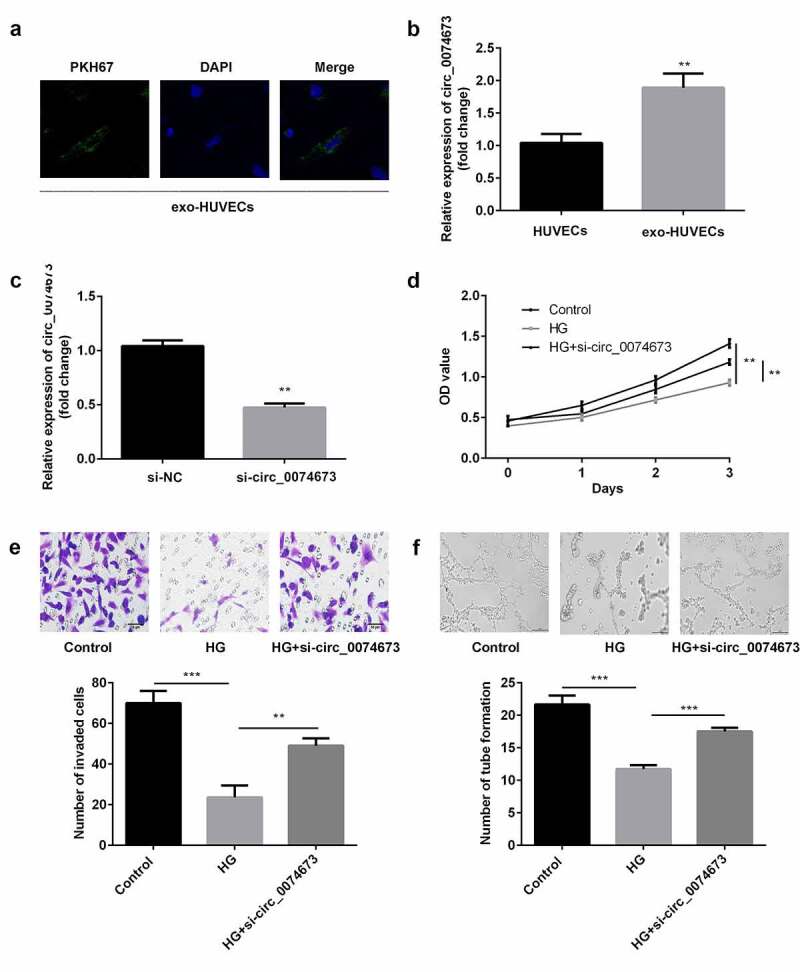
*The silence of circ_0074673 promoted the biological functions of HG-HUVECs*. (a) The exosomes were labeled using PKH67 and co-cultured with HUVECs. The images were captured using a fluorescence confocal microscope. (b) The levels of circ_0074673 in the HUVECs and exo-HUVECs were measured by qPCR. (c) Following si-circ_0074673 transfection, the level of circ_0074673 was determined by qPCR. (d) Cellular proliferation was analyzed by the CCK-8 assay. (e) Cellular migration was assessed by the transwell assay. (f) Cellular angiogenesis was analyzed using the tube formation assay. Each experiment was repeated in three times. **P < 0.01. ***P < 0.001

In order to determine whether exosomal circ_0074673 plays an essential role in the biological functions of HUVECs, the exosomes isolated from patients with GDM were incubated with HUVECs. As illustrated in Figure 2(a), the PKH67-labeled exosomes were dispersed in the HUVECs. The expression of circ_0074673 significantly increased in the exo-HUVEC group (Figure 2(b)), compared to that of the HUVEC group. The level of circ_0074673 significantly reduced following transfection with si-circ_0074673 (Figure 2(c)). The results of the CCK-8 assay revealed that HG treatment inhibited cellular proliferation. The knockdown of circ_0074673 significantly enhanced the proliferation of HG-HUVECs (Figure 2(d)). Cellular migration was significantly suppressed by HG treatment, while the knockdown of circ_0074673 remarkably promoted the migration of HG-HUVECs (Figure 2(e)). The results further demonstrated that while HG treatment decreased angiogenesis, transfection with si-circ_0074673 increased angiogenesis in the HG-HUVECs (Figure 2(f)).

### Circ_0074673 serves as an miR-1200 sponge

**Figure 3.**
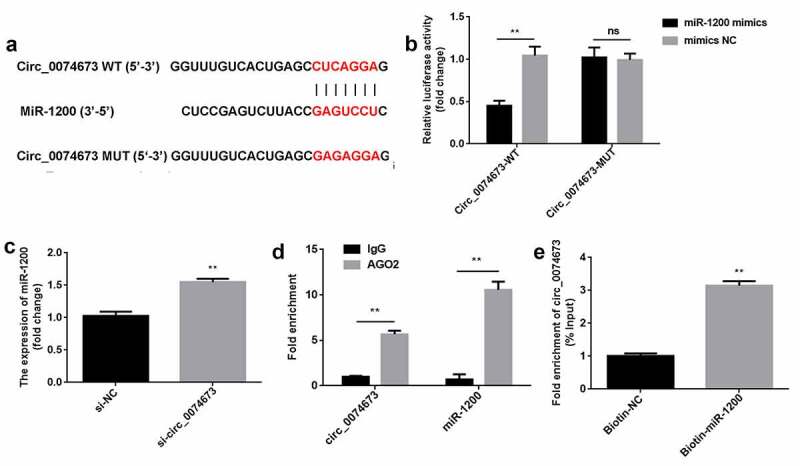
*Circ_0074673 serves as an miR-1200 sponge*. (a) MiR-1200 targets the 3′-UTR sequence of circ_004673. (b) The relative luciferase activity was determined using the luciferase reporter assay. (c) Circ_0074673 and miR-1200 co-immunoprecipitated with AGO2 was determined by RIP assay. (d) Circ_0074673 pulled-down with biotin-labeled miR-1200 by RNA pull-down assay. (e) Following si-circ_0074673 transfection, the levels of miR-1200 were detected using qPCR. Each experiment was repeated in three times. **P < 0.01. ns P > 0.05

Bioinformatic analysis revealed that circ-0074673 can directly bind to miR-1200 (Figure 3(a)). As expected, the luciferase activity significantly decreased in the circ_0074673-WT + miR-1200 mimics group, compared to that of the circ_0074673-WT + mimics NC group, while there were no significant differences in the luciferase activity of the circ_0074673-MUT + miR-1200 mimics group and circ_0074673-MUT + mimics NC group (Figure 3(b)). Additionally, anti-AGO2 binding to circ_0074673 and miR-1200 was increased, compared with anti-IgG (Figure 3(c)). Circ_0074673 was enriched in pulled-down by biotin-miR-1200 (Figure 3(d)). Moreover, the levels of miR-1200 were increased after circ_0074673 knockdown (Figure 3(e)).

### Circ_0074673 knockdown facilitated the proliferation, migration, and angiogenesis of HG-HUVECs by sponging miR-1200

**Figure 4.**
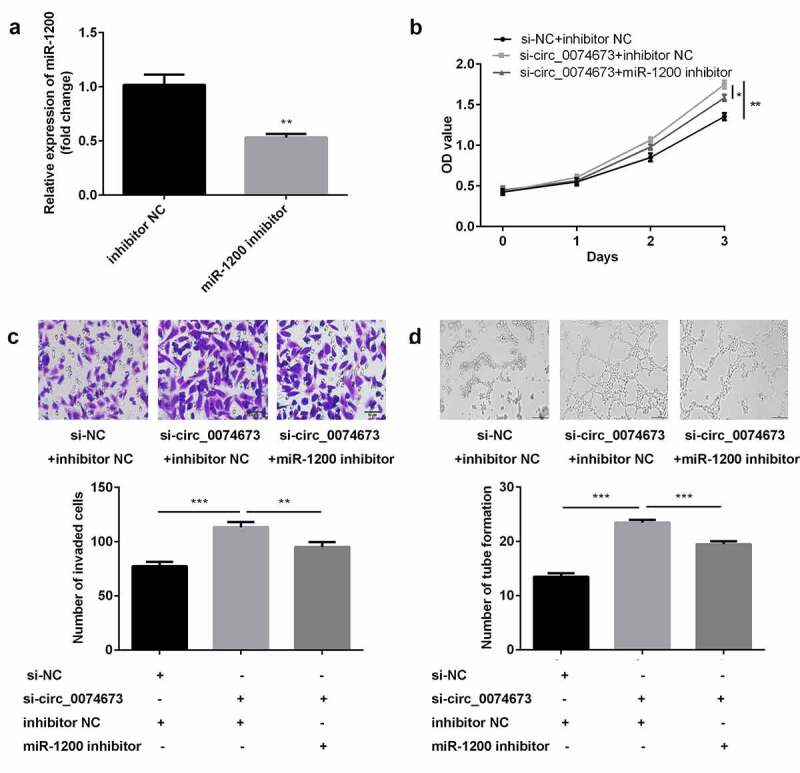
*Circ_0074673 silencing promoted the biological behaviors of HG-HUVECs by sponging miR-1200*. (a) The levels of miR-1200 were measured by qPCR following transfection with the miR-1200 inhibitor. (b) Cellular proliferation was analyzed using the CCK-8 assay. (c) Cellular migration was assessed by the transwell assay. (d) Cellular angiogenesis was analyzed by the tube formation assay. Each experiment was repeated in three times. **P < 0.01. ***P < 0.001

Following transfection with the miR-1200 inhibitor and inhibitor NC, the levels of miR-1200 were downregulated in the inhibitor-treated group (Figure 4(a)). Furthermore, the knockdown of circ_0074673 significantly promoted cellular proliferation, migration, and angiogenesis of HG-HUVECs, whereas the inhibition of miR-1200 abrogated this promotion (Figure 4(b–d)).

### MEOX2 is a downstream target of miR-1200

**Figure 5.**
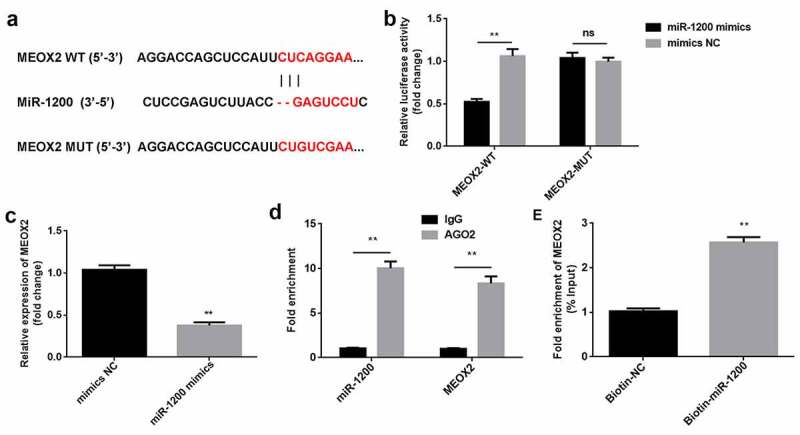
*MiR-1200 directly targets MEOX2*. (a) MiR-1200 targets the 3′-UTR region of MEOX2. (b) The relative luciferase activity was measured using the luciferase reporter assay. (c) MiR-1200 and MEOX2 co-immunoprecipitated with AGO2 was determined by RIP assay. (d) MEOX2 pulled-down with biotin-labeled miR-1200 using RNA pull-down assay. (e) The levels of MEOX2 following transfection with miR-1200 mimics were detected using qPCR. Each experiment was repeated in three times. **P < 0.01. ns P > 0.05

Bioinformatics analysis revealed that MEOX2 can directly bind to miR-1200 (Figure 5(a)). The luciferase activity was markedly reduced in the cells co-transfected with MEOX2-WT and miR-1200 mimics, compared to that of the cells treated with mimics NC. Neither the miR-1200 mimics nor the mimics NC affected the luciferase activity of the MEOX2-MUT group (Figure 5(b)). Compared with IgG, miR-1200 and MEOX2 could bind to AGO2 (Figure 5(c)). Moreover, MEOX2 was enriched in pulled-down by biotin-miR-1200 (Figure 5(d)). As illustrated in Figure 5(e), the overexpression of miR-1200 downregulated the expression of MEOX2.

### MiR-1200 overexpression facilitated the proliferation, migration, and angiogenesis of HG-HUVECs by targeting MEOX2

**Figure 6.**
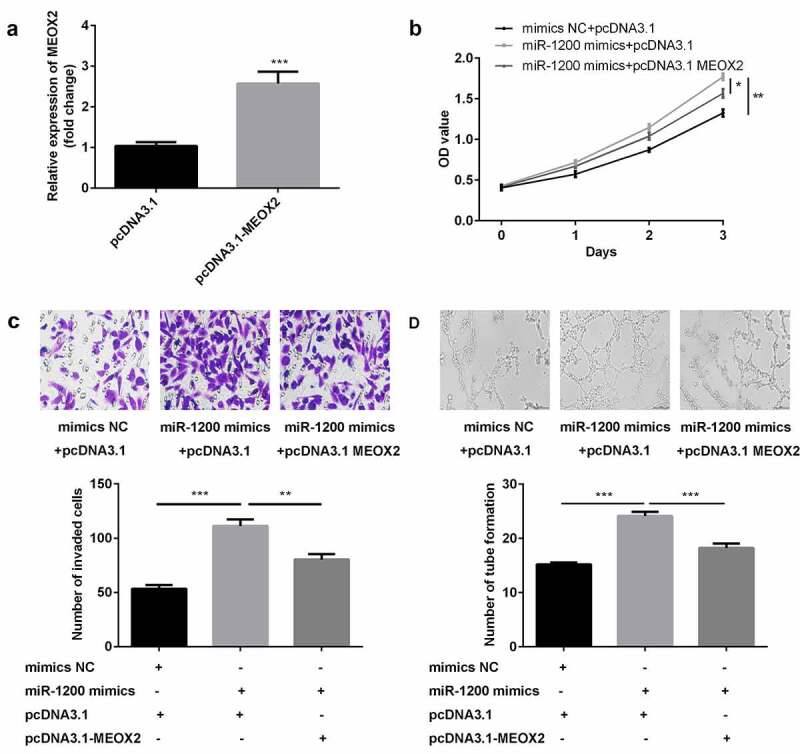
*The overexpression of miR-1200 promoted the biological functions of HG-HUVECs by targeting MEOX2*. (a) The levels of MEOX2 were measured by qPCR following transfection with pcDNA3.1-MEOX2. (b) Cellular proliferation was analyzed by the CCK-8 assay. (c) Cellular migration was assessed by the transwell assay. (d) Cellular angiogenesis was analyzed using the tube formation assay. Each experiment was repeated in three times. **P < 0.01. ***P < 0.001

The results of studies on transfection efficiency revealed that the levels of MEOX2 were upregulated in cells transfected with pcDNA3.1 (Figure 6(a)). Furthermore, cellular proliferation, migration, and angiogenesis of HG-HUVECs were promoted by the overexpression of miR-1200, whereas the MEOX2 abrogated this promotion (Figure 6(b–d)).

## Discussion

In this study, the expression of circ_0074673 was upregulated in the exosomes isolated from the umbilical cord blood of patients with GDM and in the exo-HUVECs. Moreover, the silencing of exosomal circ_0074673 promoted the biological functions of HUVECs by regulating the miR-1200/MEOX2 axis.

We successfully isolated exosomes from the umbilical cord blood of patients with GDM and the healthy controls according to the results of exosomes size, concentration and the CD9 and CD63 levels. And the results of the PKH67 staining assay showed exosomes are dispersed in the HUVECs.

Accumulating circRNAs have been reported to have potential regulatory roles in GDM [22]. For instance, a previous study demonstrated that the knockdown of circ_0005243 suppresses the proliferation and migration of trophoblast cells [23]. Circ_0008285 is related to increased glycated hemoglobin, and its knockdown alleviates the progression of GDM [24]. Previous studies have reported that GDM is associated with endothelial dysfunction [8,9]. Recent studies have demonstrated that circRNAs partake in the regulation of angiogenesis [25]. The results of this study revealed that the expression of exosomal circ_0074673 was upregulated in patients with GDM. We therefore focused on investigating the role of circ_0074673 in HUVECs. We observed that the levels of circ_0074673 increased in the exosomes isolated from the umbilical cord blood of patients with GDM and the exosomes co-cultured with HUVECs. Furthermore, the knockdown of circ_0074673 facilitated the proliferation, migration, and angiogenesis of HG-HUVECs. These findings suggested that the silencing of exosomal circ_0074673 attenuates the progression of GDM.

A large number of circRNAs exert biological functions by acting as miRNA sponges [11]. The results of this study revealed that circ_0074673 acts as a miR-1200 sponge. To date, few studies have investigated the expression of miR-1200. It has been reported that miR-1200 acts as a diagnostic and/or prognostic marker for tumors [26]. Previous studies have demonstrated that miR-1200 partakes in tumor progression through regulation by the long noncoding RNA (lncRNA) RGMB-AS1 and circ_0001785 [27,28]. It has been additionally reported that miR-1200 is associated with drug resistance [29]. In GDM, the upregulation of CCND2 is negatively regulated by miR-1200 [30]. The results of this study demonstrated that miR-1200 was negatively regulated by circ_0074673. Furthermore, the downregulation of miR-1200 abolished the effects of circ_0074673 knockdown on the cellular functions of HG-HUVECs. These results suggested that the silencing of circ_0074673 attenuated the progression of GDM by sponging miR-1200.

MEOX2, also known as GAX, is a transcription factor that is expressed in nearly all vascular smooth muscle cells and endothelial cells [31]. MEOX2 is a regulator of the angiogenic phenotype and inhibits cellular growth and tube formation [32]. Additionally, the increased expression of MEOX2 is associated with the senescence of endothelial cells [33]. A previous study revealed that MEOX2 is upregulated in pregnant women with GDM, which promotes the migration of endothelial colony-forming cells [34]. In the present study, MEOX2 was identified as a target of miR-1200. Moreover, the overexpression of MEOX2 abrogated the effects of miR-1200 on the biological functions of HG-HUVECs, suggesting that miR-1200 attenuated the development of GDM by targeting MEOX2.

However, this study has potential limitations. The first is the sample of the subjects is small. Additionally, the role of circ_0074673 *in vivo* is still known. We will study in future.

## Conclusion

This study is the first to investigate the roles of exosomal circ_0074673 in HUVECs. The knockdown of exosomal circ_0074673 facilitated the proliferation, migration, and angiogenesis of HG-HUVECs by regulating the miR-1200/MEOX2 axis. These findings suggested that exosomal circ_0074673 might be a potential therapeutic target for GDM.

## Highlights

1. The expression of circ_0074673 is increased in exosome from patients with GDM.

2. Knockdown of exosomal circ_0074673 promotes the biological behavior of HG-HUVECs.

3. Exosomal circ_0074673 and MEOX2 directly bind to miR-1200.

4. Exosomal circ_0074673 regulates the biological behavior of HG-HUVECs by the miR-1200/MEOX2 axis.
